# CD301b^+^ macrophage: the new booster for activating bone regeneration in periodontitis treatment

**DOI:** 10.1038/s41368-023-00225-4

**Published:** 2023-05-17

**Authors:** Can Wang, Qin Zhao, Chen Chen, Jiaojiao Li, Jing Zhang, Shuyuan Qu, Hua Tang, Hao Zeng, Yufeng Zhang

**Affiliations:** 1 The State Key Laboratory Breeding Base of Basic Science of Stomatology (Hubei- MOST) & Key Laboratory of Oral Biomedicine, Ministry of Education, School & Hospital of Stomatology, Wuhan University, Wuhan, China; 2 Institute of Infection and Immunity, Science and Technology Innovation Center, Shandong First Medical University & Shandong Academy of Medical Sciences, Jinan, Shandong PR China; 3 Medical Research Institute, School of Medicine, Wuhan University, Wuhan, China; 4 Taikang Center for Life and Medical Sciences, Wuhan University, Wuhan, China

**Keywords:** Tissue engineering, Periodontitis

## Abstract

Periodontal bone regeneration is a major challenge in the treatment of periodontitis. Currently the main obstacle is the difficulty of restoring the regenerative vitality of periodontal osteoblast lineages suppressed by inflammation, via conventional treatment. CD301b^+^ macrophages were recently identified as a subpopulation that is characteristic of a regenerative environment, but their role in periodontal bone repair has not been reported. The current study indicates that CD301b^+^ macrophages may be a constituent component of periodontal bone repair, and that they are devoted to bone formation in the resolving phase of periodontitis. Transcriptome sequencing suggested that CD301b^+^ macrophages could positively regulate osteogenesis-related processes. In vitro, CD301b^+^ macrophages could be induced by interleukin 4 (IL-4) unless proinflammatory cytokines such as interleukin 1β (IL-1β) and tumor necrosis factor α (TNF-α) were present. Mechanistically, CD301b^+^ macrophages promoted osteoblast differentiation via insulin-like growth factor 1 (IGF-1)/thymoma viral proto-oncogene 1 (Akt)/mammalian target of rapamycin (mTOR) signaling. An osteogenic inducible nano-capsule (OINC) consisting of a gold nanocage loaded with IL-4 as the “core” and mouse neutrophil membrane as the “shell” was designed. When injected into periodontal tissue, OINCs first absorbed proinflammatory cytokines in inflamed periodontal tissue, then released IL-4 controlled by far-red irradiation. These events collectively promoted CD301b^+^ macrophage enrichment, which further boosted periodontal bone regeneration. The current study highlights the osteoinductive role of CD301b^+^ macrophages, and suggests a CD301b^+^ macrophage-targeted induction strategy based on biomimetic nano-capsules for improved therapeutic efficacy, which may also provide a potential therapeutic target and strategy for other inflammatory bone diseases.

## Introduction

Periodontitis is a chronic inflammatory disease characterized by periodontal bone destruction and eventual tooth loss. Restoring periodontal bone height and salvaging teeth is a significant challenge. The uncoupling of bone formation from resorption caused by inflammation accounts for periodontal bone loss.^1^ Therefore, researchers have focused on regulating immune cells to reduce inflammation in the tissue microenvironment, inhibiting osteoclast formation and promoting periodontal bone repair.^2–4^ Notably however, clinical patient-based single-cell sequencing studies of periodontal tissue indicate that there are associated problems such as depletion of osteoblast lineages and insufficient bone regenerative vitality after periodontal treatment.^5^ There is therefore an urgent need to identify a “booster” in periodontal tissues that can directly and potently regulate the osteogenic lineage, and facilitate the development of new immunomodulatory therapeutic strategies.

As sentinels of the body’s immune response, macrophages are essential for resisting bacterial infections, regulating immune responses, and mediating inflammatory tissue damage and repair.^6^ Traditionally scholars have divided macrophages into classically activated macrophages (M1) and alternatively activated macrophages (M2), and have attempted to promote the polarization of macrophages to an M2 phenotype in various ways, to reduce inflammation and create a suitable immune microenvironment for tissue regeneration.^7–9^ The M1/M2 classification is primarily based on the ability to modulate inflammation rather than the ability to promote regeneration. Macrophages exhibit extensive heterogeneity, and it has been reported that they can coordinate fracture repair and healing in mice by secreting low-density lipoprotein receptor-related protein 1, but the precise subpopulation involved has not been identified.^10,11^ Sommerfeld et al.^12^ reported that CD301b^+^ macrophages were specific for the tissue regeneration environment, rather than CD206^+^ macrophages or CD86^+^ macrophages. In another recent study adoptive transfer of CD301b^+^/CD206^+^ macrophages achieved better skin regeneration compared to CD301b^-^/CD206^+^ macrophages.^13,14^ These observations suggest that CD301b^+^ macrophages may be a “pro-regenerative” population distinct from M2 macrophages. However, the effects of CD301b^+^ macrophages on periodontal bone repair have not been reported.

With the in-depth study of immune responses and inflammation by scholars, tissue engineering technology based on immune regulation has gradually become an effective means to treat inflammatory diseases and promote tissue regeneration.^15,16^ Immunomodulators have been used to reduce inflammation and foster an immune microenvironment suitable for tissue regeneration. Among them, immunomodulatory nanomaterials—particularly biomimetic nanomaterials coated with immune cell membranes—can evidently provide potent support in the treatment of inflammatory diseases and the promotion of bone regeneration.^17^ Native protein markers on the outer surface can equip nanoparticles with adaptive and regulatory functions such as prolonged blood circulation, antigen recognition, enhanced targeting, better interaction with surrounding cells, and reduced toxicity in vivo, all of which make up for the deficiencies of nanomaterials.^17,18^ For example, macrophage-coated nanomaterials have been used to control inflammation and promote healing and repair of bone defects.^19^ Notably however, immunomodulatory strategies for periodontitis are mainly aimed at enriching anti-inflammatory immune cells such as M2 and regulatory T cells.^20,21^ Herein we propose a new research paradigm focused on identifying macrophage subsets that have direct positive regulatory effects on osteoblast lineages, and targeting them to enhance osteoblast lineage activity and promote periodontal bone regeneration.

In the murine periodontitis model we observed bone loss and regeneration during the progressive and resolving phases of periodontal inflammation. The role of CD301b^+^ macrophages in periodontal bone repair was investigated during the resolution of inflammation. Transcriptome sequencing and in vitro experimental functional validation revealed specific mechanisms of positive effects on bone mesenchymal stem cell (BMSC) osteogenic differentiation. Further, we designed CD301b^+^ macrophage-targeted mimetic osteoinductive nano-capsules to achieve better promotion of periodontal bone regeneration. The current study reveals the regenerative potential of CD301b^+^ macrophages in periodontal therapy, and demonstrates a therapeutic application which may constitute a prospective basis for use in other bone regeneration scenarios.

## Results

### Restored bone formation after resolution of periodontitis

**Fig. 1:**
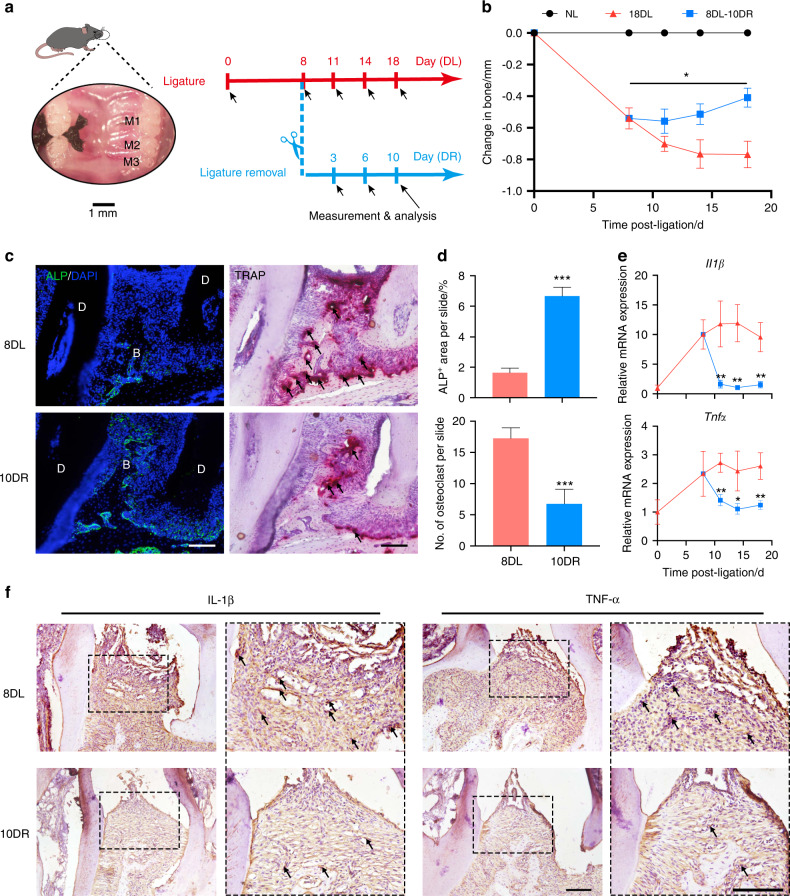
Periodontal bone exhibits a degree of repair after periodontal inflammation subsides. **a** Schematic illustration of a murine periodontitis model establishment in the inflammatory and healing stages with ligature persistence and removal. M1–3, the three molars. **b** Bone level changes on the buccal side during the inflammatory and healing phases of periodontitis were calculated relative to the non-ligated (NL) contralateral site (baseline) (*n* = 4). **c** Representative images of immunofluorescent staining for ALP and TRAP on periodontal tissue sections from the 8DL and 10DR groups. D dentin, B bone. Scale bar, 100 μm. **d** Quantification of ALP-positive area and TRAP-positive osteoclast number on sagittal sections from the 8DL and 10DR groups (*n* = 4). **e** Alterations in mRNA expression of the inflammatory cytokines IL-1β and TNF-α in periodontal lesions from different groups. Data were normalized to GADPH mRNA and are presented as fold change relative to baseline, set as 1 (*n* = 4). **f** Representative immunohistochemistry micrographs of 8DL and 10DR groups identifying IL-1β and TNF-α. Arrows indicate positive cells in the slide. Scale bar, 100 μm

A murine periodontitis model was established via silk ligation and ligature removal, and the associated dynamic bone changes were observed (Fig. 1a). At day 8 of ligation (8DL) significant bone loss was induced, indicating successful establishment of the model (Fig. S1). Within 10 days after ligature removal (10DR) bone resorption was reversed and bone height gradually began to increase, whereas bone level continued to decline in mice subjected to another 10 days of constant ligation (Fig. S1, Fig. 1b). Consistently, compared with the 8DL group, periodontal tissue in the 10DR group exhibited higher alkaline phosphatase (ALP) activity and reduced osteoclast (tartrate-resistant acid phosphatase positive, TRAP^+^) numbers (Fig. 1c, d). To investigate periodontal inflammation status the expression levels of two critical periodontitis-related inflammatory factors were examined at different timepoints of ligature persistence and removal, IL-1β and TNF-α. In the removal group these proinflammatory cytokines were expressed at lower levels than in the ligation group (Fig. 1e). Consistent results were obtained via immunohistochemical staining, in which the cells of positive IL-1β and TNF-α staining were significantly decreased after ligature removal (Fig. 1f). These findings indicate that stimulus elimination in murine periodontitis could promote inflammation resolution, followed by restored bone formation to an extent.

### Altered macrophage activation is associated with periodontal bone formation

**Fig. 2:**
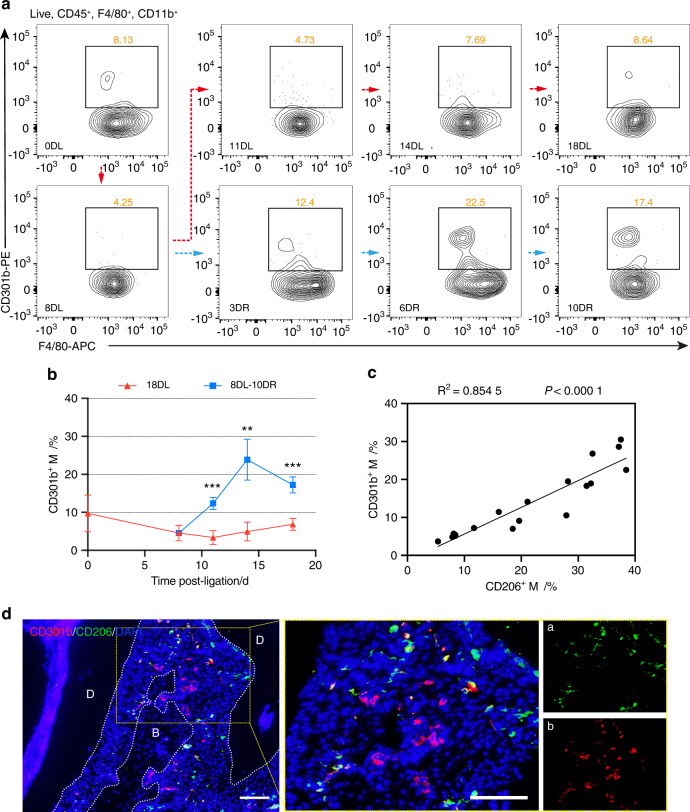
CD301b^**+**^ macrophages are enriched at the frontier of periodontal bone repair. **a** Representative flow cytometry contour plots of CD11b^+^F4/80^+^CD301b^+^ macrophages (Φ) throughout the progression and healing stages of periodontitis. **b** Quantification of the percentage of CD301b^+^ macrophages within periodontal lesions at indicated timepoints (*n* = 3). **c** Correlations between the percentages of CD301b^+^ and CD206^+^ macrophages at different timepoints. **d** Immunofluorescent staining of periodontal tissue sections from the 6DR group to identify the locations of CD301b^+^CD206^+^, CD301b^+^CD206^-^, and CD301b^-^CD206^+^ macrophages. The yellow frame indicates the locally amplified area. D dentin, B bone. Scale bar, 100 μm

To further characterize changes in macrophage phenotypes associated with different periodontal inflammatory states, flow cytometry was used to analyze the dynamics of different subpopulations via the gating strategy shown in Fig. S2a. Three major subsets of macrophages in periodontal tissue were evident; CD11b^+^F4/80^+^Ly6C^-^CD206^+^ macrophages (denoted as the anti-inflammatory macrophage, M2), CD11b^+^F4/80^+^Ly6C^+^CD206^-^ macrophages (denoted as the inflammatory macrophages, IM), and CD11b^+^F4/80^+^CD301b^+^ macrophages (denoted as CD301b^+^ macrophages). M2 macrophages were enriched in the ligature removal phase, whereas IM macrophages were considerably more abundant in the ligature persistence phase (Fig. S2b). The percentage of M2 macrophages was inversely correlated with that of IM macrophages during the progression and healing phases of periodontitis (Fig. S2c). CD301b^+^ macrophages were more abundant in the resolving phase and peaked on day 6 after ligature removal (6DR), whereas comparatively few were present in the inflammatory phase (Fig. 2a, b). Alteration of M2 macrophages correlated strongly with that of CD301b^+^ macrophages (Fig. 2c). Within the CD301b^+^ macrophage pool, 7.94% were CD206^+^ and 11.00% were CD206^-^ in the 10DR group, whereas 1.66% were CD206^+^ and 3.19% were CD206^-^ in the 8DL group (Fig. S3). Hence, within periodontal macrophages, the CD301b^+^ subset and the CD206^+^ subset overlapped to an extent, but were not an identical population. Immunofluorescence analysis showed that compared with CD206^+^ macrophages, CD301b^+^ macrophages accumulated at the frontier of periodontal bone repair (Fig. 2d). Therefore, CD301b^+^ macrophages may be a novel subset distinct from M2 macrophages, and may be closely associated with bone repair.

### CD301b^+^ macrophages are essential for bone formation during the resolution phase

**Fig. 3:**
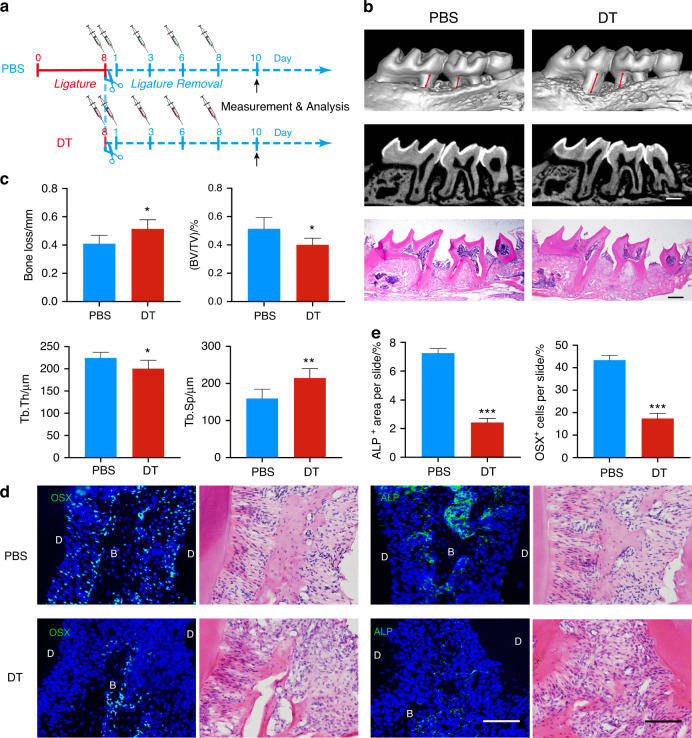
CD301b^**+**^ macrophages are required for periodontal bone repair. **a** The study protocol of periodontitis model establishment in *Mgl2*^DTR^ mice injected with PBS or DT to investigate periodontal bone changes during the resolving phase. **b** Representative images of μCT analysis and H&E staining to observe bone loss (bidirectional red arrows) in the DT and PBS group. Scale bar, 500 μm. **c** Based on three-dimensional μCT reconstruction, bone loss and other relevant bone parameters (BV/TV, Tb.Th, Tb.Sp) are quantified (*n* = 4). **d** Immunofluorescence staining of the osteogenic marker OSX and ALP in periodontal tissue sections from CD301b^+^ macrophage-depleted and non-depleted groups followed by corresponding H&E staining. D dentin, B bone. Scale bar, 100 μm. **e** Quantitative analysis of the proportion of OSX^+^ cells and the proportion of ALP^+^ areas in two groups (*n* = 3)

To further investigate the function of CD301b^+^ macrophages in the resolving phase of periodontitis, diphtheria toxin (DT) was administered to *Mgl2*^DTR^ mice (Fig. S4a) at corresponding timepoints to deplete CD301b^+^ macrophages (Fig. 3a). Compared with phosphate-buffered saline (PBS), DT administration significantly reduced the proportion of periodontal CD301b^+^ macrophages to a quarter in *Mgl2*^DTR^ mice, suggesting the successful depletion of CD301b^+^ macrophages with high efficiency (Fig. S4b). Micro-computed tomography (μCT) analysis and corresponding hematoxylin and eosin (H&E) staining revealed less bone formation after CD301b^+^ macrophage depletion (Fig. 3b). Other bone parameters such as bone loss, bone volume/tissue volume (BV/TV), trabecular thickness (Tb.Th.), and trabecular separation (Tb.Sp.) exhibited the same results (Fig. 3c). Reduced expression of osteoblast markers such as ALP and Osterix (OSX) were observed in the DT group (Fig. 3d, e). Expression levels of inflammatory cytokines such as IL-1β and TNF-α were not influenced by CD301b^+^ macrophage depletion (Fig. S5). Overall, these data indicated that CD301b^+^ macrophages were required for bone formation in the resolving period but not for inflammation resolution.

### CD301b^+^ macrophage-derived IGF-1 promotes osteoblast differentiation

**Fig. 4:**
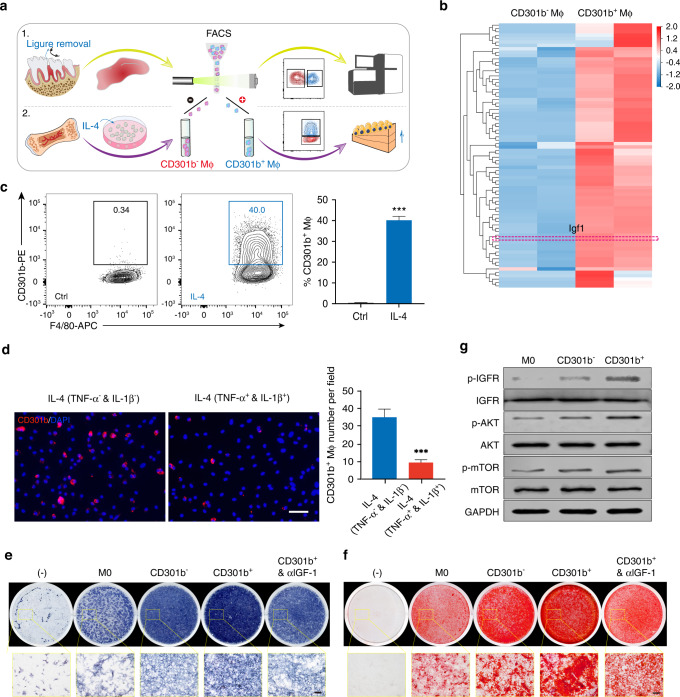
CD301b^**+**^ macrophage-derived IGF-1 promotes osteogenic differentiation via the IGFR/AKT/mTOR pathway. **a** Study design illustrating the sorting of CD301b^+^ macrophages from periodontal tissue in vivo for RNA-Seq assays, and from IL-4-induced BMDMs in vitro for cellular experiments. **b** Heatmap of the top 78 upregulated genes associated with regulation of osteogenesis in the GO analysis. **c** Flow cytometry analysis and quantification of CD301b^+^ macrophages with or without IL-4 induction of BMDMs for 24 h (*n* = 3). **d** Cellular immunofluorescence for CD301b in IL-4-activated BMDMs incubated with or without IL-1β and TNFα for 24 h. The number of CD301b^+^ macrophages per field was quantified (*n* = 3). **e, f** ALP and alizarin red staining to reveal the effects of CM collected from different groups on the osteogenic induction of BMSCs for 7 and 14 days. **g** Western blotting detection of p-IGFR, IGFR, p-Akt, Akt, p-mTOR, and mTOR in BMSCs with different treatments

To characterize the nature of CD301b^+^ macrophages, the transcriptomes of CD301b^+^ macrophages and CD301b^−^ macrophages isolated from periodontal tissues at 6DR were compared (Fig. 4a1). Among the differentially expressed genes (DEGs), gene ontology (GO) enrichment analysis and gene set enrichment analysis (GSEA) suggested that several osteogenesis-related processes such as ossification and osteoblast differentiation—rather than suppressing the inflammatory response—were positively regulated by CD301b^+^ macrophages (Fig. S6a, b). Seventy-eight upregulated genes associated with the promotion of osteogenesis-related processes (*e.g*., “positive regulation of cell proliferation”, “regulation of bone mineralization”, and “positive regulation of osteoblast differentiation”) were evident in a heat map, among which the expression of *igf1* was significantly upregulated (Figs. S6a, b and 4b). The expression level of *igf1* in periodontal tissues was significantly reduced in the CD301b^+^ macrophage depletion group at 10DR (Fig. S7a). These data indicated that CD301b^+^ macrophage-derived IGF-1 may play an important role in periodontal bone repair.

Given the limited number of CD301b^+^ macrophages obtained from periodontal tissues, CD301b^+^ macrophages were induced in vitro to investigate their effects on the osteogenic differentiation of BMSCs. Bone marrow-derived macrophages (BMDMs) were incubated with IL-4 and induced ~40% CD301b^+^ macrophages (Fig. 4c). However, in the presence of IL-1β and TNF-α the induction efficiency was reduced to a quarter (Fig. 4d). This result was consistent with the decreased proportion of CD301b^+^ macrophages in the inflammatory phase of periodontal tissue.

To investigate the functions and mechanisms associated with CD301b^+^ macrophages in osteoblast differentiation, CD301b^−^ and CD301b^+^ macrophages were sorted from IL-4-incubated BMDMs, and culture supernatants were collected as conditioned medium (CM) for further research (Fig. 4a2). Consistent with the in vivo transcriptome sequencing results, *igf1* expression was significantly elevated in in vitro-induced CD301^+^ macrophages relative to CD301b^-^ macrophages and untreated BMDMs (M0) (Fig. S7b). ALP and alizarin red staining showed that CD301b^+^ macrophages obviously increased the osteoblast differentiation of BMSCs compared with CD301b^-^ macrophages and M0. However, CD301b^+^ macrophage-mediated promotion could be counteracted by IGF1-neutralizing antibody (αIGF-1) (Fig. 4e, f). The expression of osteogenesis-related genes such *as runt-related transcription factor 2 (runx2)*, *alp*, and *collagen-I (col1)* also proved the same phenomenon (Fig. S8). To investigate the mechanisms involved in IGF-1-mediated promotion of osteogenic differentiation, phosphorylation levels of IGFR, Akt, and mTOR were detected. CD301b^+^ macrophage-derived IGF-1 stimulated the phosphorylation of IGFR, Akt, and mTOR in BMSCs (Fig. 4g).

### Design, characterization, and functional verification of osteogenic inducible nano-capsules

**Fig. 5:**
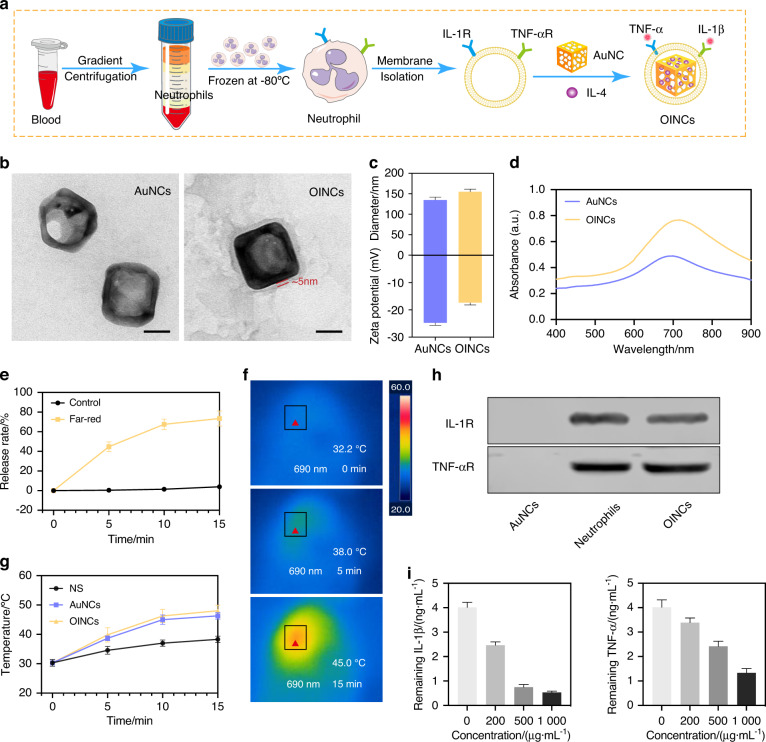
Design, characterization, and functional verification of OINCs. **a** Pattern diagram of the design and synthesis of OINCs. **b** Representative transmission electron microscopy image of AuNC and OINC. Scale bar, 50 nm. **c** Dynamic light scattering measurements indicating hydrodynamic size and zeta potential of AuNCs and OINCs. **d** Ultra-violet-visible absorption spectra of AuNCs and OINCs under different wavelengths of irradiation. **e** Release rate curve of OINCs with or without far-red irradiation. **f**, **g** Representative photothermal images of OINCs injected into periodontal tissue under 690-nm far-red irradiation, and temperature-time curves of AuNCs and OINCs compared with the normal saline group. **h** Characteristic protein bands of AuNCs, neutrophil lysates, and OINCs resolved by western blotting. **i** Binding capacity of different concentrations of OINCs with IL-1β and TNF-α

Cell-mimicking nano-capsules designed to promote bone regeneration in periodontitis were generated. Gold nanocages (AuNCs) were synthesized in accordance with a previously described protocol.^22^ The cell counting kit-8 assay (CCK-8) revealed no effects on cell viability when the concentration of AuNCs was ≤50 μg·mL^−1^ (Fig. S9). Osteogenic inducible nano-capsules (OINCs) composed of mouse neutrophil membrane as the “shell” and IL-4-loaded AuNCs as the “core” were then synthesized via the extrusion method (Fig. 5a).

Transmission electron microscopy (TEM) depicted a square core shell structure wrapped by a uniform membrane with a thickness of ~5 nm (Fig. 5b). Dynamic light scattering measurements revealed that the hydrodynamic diameter of OINCs increased by ~15 nm compared with that of AuNCs, whereas the surface zeta potential of OINCs was reduced (Fig. 5c). The ultra-violet-visible absorption peaks of AuNCs and OINCs were both at ~690 nm (Fig. 5d). The photothermal effect of OINCs was similar to that of AuNCs (Fig. S10). The drug loading efficiencies of AuNCs was ~9.12%, and the encapsulation efficiencies were ~73.59% as previously reported.^23^ The photo-controlled release curve of drug-loaded AuNCs was similar to the photothermal curves, demonstrating that after in vitro irradiation for 3 min the temperature of AuNCs rose to ~38 °C, corresponding to drug release of ~25%. For 5 min the temperature increased to ~45 °C, corresponding to ~45% drug release (Fig. 5e and S10). Because the photothermal curve of AuNCs applied in vivo differed slightly from that in vitro, the photothermal curve of AuNCs and OINCs in periodontal tissue was investigated. Compared with the normal saline group, the photothermal curves of AuNCs and OINCs in vivo were similar while the heating efficiency was lower than that of in vitro. For example, irradiation for 5 min only raised the temperature to 38 °C in vivo, which corresponded to 25% drug release (Fig. 5f, g).

TNF-α receptor (TNF-αR) and IL-1β receptor (IL-1R) were confirmed as present on OINCs, suggesting the successful translocation of mouse neutrophil membrane onto the AuNC core (Fig. 5h). The capacity of OINCs to absorb IL-1β and TNF-α was then tested via enzyme-linked immunosorbent assays (ELISAs). OINCs containing 500 μg·mL^−1^ membrane protein exhibited removal efficiencies of 78.0% for IL-1β binding and 40.0% for TNF-α binding. OINCs containing 1 000 μg·mL^−1^ membrane protein exhibited removal efficiencies of 87.5% for IL-1β binding and 67.5% for TNF-α binding (Fig. 5i). Thus, OINCs could effectively absorb IL-1β and TNF-α in a concentration-dependent manner.

### OINCs enhance periodontal bone regeneration by inducing the formation of CD301b^+^ macrophages

**Fig. 6:**
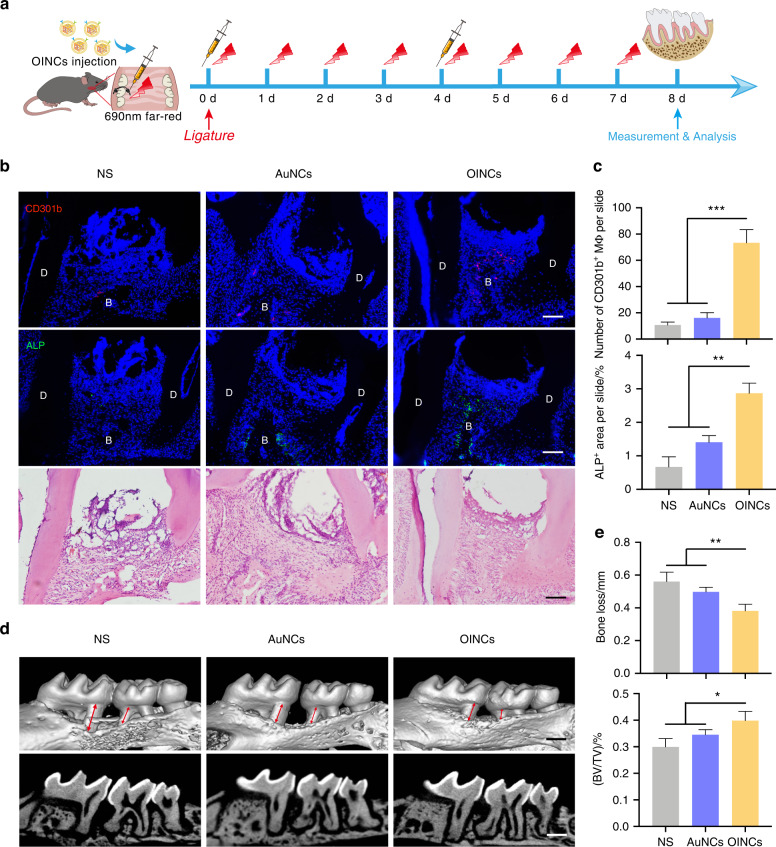
OINCs promote periodontal bone regeneration by inducing the formation of CD301b^**+**^ macrophages. **a** Flowchart of the time-course of OINC administration and irradiation in a mouse periodontitis model. **b** Representative images of immunofluorescent staining for CD301b^+^ macrophages and ALP, and corresponding H&E staining in the different groups including normal saline (NS), AuNCs, and OINCs. **c** Quantitative analysis of CD301b^+^ macrophage number and ALP^+^ area per slide (*n* = 3). **d** μCT analysis and Masson staining to compare levels of regenerative bone in different groups. **e** Quantification of bone-associated parameters (bone loss, BV/TV) to evaluate the therapeutic efficacy of OINCs (*n* = 4)

The efficacy of OINCs for promoting periodontal bone regeneration via the absorption of inflammatory factors and CD301b^+^ macrophage induction was evaluated in murine periodontitis. After ligature placement between the first and second molar, periodontal lesions were micro-injected with OINCs, and mice were injected with AuNCs or normal saline served as control. The schedule of injections and irradiations is shown in Fig. 6a. Immunofluorescent staining imaging indicated that after OINCs injection CD301b^+^ macrophages increased markedly in periodontal tissue compared with the normal saline and AuNC groups. Expression of the vital osteogenic marker ALP was also highly upregulated in the OINC group, suggesting boosted osteogenic function. H&E staining indicated reduced immune cell infiltration (Fig. 6b, c). μCT analysis revealed higher bone levels and bone mass in the OINC group than in the normal saline and AuNC groups (Fig. 6d, e). Hence, the biomimetic nano-capsule system with absorption of inflammatory factors and controlled release of IL-4 can efficiently induce CD301b^+^ macrophages, which further boosts periodontal bone formation.

## Discussion

Periodontitis is prevalent worldwide, and is the primary cause of tooth loss in adults. Given that bacteria initiate periodontitis, and inflammation is the main cause of periodontal bone destruction, traditional treatments are mainly aimed at removing pathogenic bacteria and reducing periodontal inflammation.^24^ Notably however, a recent study indicated that after conventional periodontal therapy there are problems of osteogenic lineage depletion and insufficient osteogenic activity that echo the irreversible bone loss associated with clinical periodontitis.^5,25^ Therefore, how to activate the vitality of the osteoblast lineage and restore periodontal bone height is a critical challenge to be overcome.

In the current study a periodontitis model was established in mice, and after ligature removal the level of inflammation was reduced and periodontal tissue facilitated a degree of bone formation, which was consistent with a previous study.^26^ To identify subpopulations of macrophages that were closely associated with inflammatory status and dynamic changes in periodontal bone height, we examined the proportions of IM macrophages, M2 macrophages, and CD301b^+^ macrophages via flow cytometry. Research indicates that IM macrophages can amplify tissue inflammatory processes, induce phagocytosis, and release several inflammatory mediators such as IL-1β and TNF-α.^27–30^ Unsurprisingly, flow cytometry analysis indicated that the proportion of IM macrophages increased in periodontal tissue in the inflammatory phase, whereas the proportion of M2 macrophages increased in the resolving phase.

The proportion of CD301b^+^ macrophages increased with periodontal bone formation and the resolution of inflammation. CD301b^+^ macrophages have traditionally been considered M2 macrophages, but recent research suggests that not all CD301b^+^ macrophages in adipose tissue express CD206, and some express an intermediate amount of the M1 marker CD11c, blurring the M1/M2 macrophage demarcation line.^31,32^ We also found that CD301b^+^ macrophages and CD206^+^ macrophages only partially overlapped in periodontal tissues, further suggesting that CD301b^+^ macrophages are a special group that do not belong to the M1/M2 classification.

The proportions of CD301b^+^ macrophages and CD206^+^ macrophages in different phases of periodontitis altered in a consistent manner, which was similar to reported observations in studies on skin healing.^13,14^ The strong correlations between the dynamic changes in two subpopulations may be inextricably associated with the strong associations between their functional roles. Further, immunofluorescence colocalization results revealed that CD301b^+^ macrophages were more closely associated with the site of bone repair than CD206^+^ macrophages. The adjacent spatial location of macrophages often suggests a specific regulatory function, and Ratnayake et al. ^33^ reported that “dwelling” macrophages adjacent to injured muscle could enhance muscle regeneration via the secretion of niche signals such as nicotinamide phosphoribosyltransferase. To further investigate whether CD301b^+^ macrophages affect periodontal bone repair by regulating inflammation, we exclusively depleted CD301b^+^ macrophages and found that periodontal bone repair was attenuated but the level of inflammation was not affected. This again suggested that CD301b^+^ macrophages may be a subpopulation that promotes periodontal bone repair independent of inflammatory regulation.

To investigate the exact role of CD301b^+^ macrophages in periodontal bone formation, macrophages were sorted in the resolving phase, and transcriptome sequencing analysis showed that CD301b^+^ macrophages have a bone repair-promoting phenotype and express higher levels of genes related to the regulation of osteogenic differentiation such as *Tgfβ1, Pdgfc*, and *Igf-1*. Given that IGF-1 was one of the most important growth factors deposited in the bone matrix and was essential for bone remodeling,^34,35^ we hypothesized that CD301b^+^ macrophages may promote periodontal bone repair by secreting IGF-1. Previous studies indicate that *mgl2* (CD301b) expression is downstream of signal transducers and activators of transcription 6 in response to IL-4 stimulation.^36^ A recent study also found a high level of IL4ra expression on CD301b^+^ macrophages.^12^ These observations are consistent with our findings. GO analysis suggests that IL-4 may be involved in the induction of CD301b^+^ macrophages. In vitro experiments confirmed that CD301b^+^ macrophage-derived IGF-1 plays an important role in osteogenic differentiation of BMSCs. However, the osteogenesis-promoting effect of CD301b^+^ macrophages may not be limited to the secretion of IGF-1. For example, our group previously demonstrated that CD301b^+^ macrophages can promote angiogenesis by secreting vascular endothelial growth factor.^37^ To validate the mechanism of IGF-1-induced differentiation of BMSCs, we focused on IGFR/Akt/mTOR signaling because it is the sensor of IGF-1 that regulates bone formation by coordinating the proliferation and osteogenic differentiation of BMSCs.^35,38^ The results indicated that activated Akt/mTOR signaling is responsible for the role of CD301b^+^ macrophages in the promotion osteogenic differentiation.

With the rapid development of biomaterials and tissue engineering technology, researchers have focused on the treatment of periodontitis and the regeneration of periodontal bone. Zhang et al. ^39^ designed an immunomodulatory microneedle patch facilitating IL-4 and TGF-β delivery to induce the repolarization of M2 macrophages and the formation of regulatory T cells. This therapeutic effect related to IL-4 release primarily aims to reduce periodontal inflammation and inhibit osteoclast function. Nanomaterials loaded with lipoxin analog also have similar periodontal bone protection effects.^40^ Considering that periodontal pathogenic bacteria and periodontal inflammation are difficult to completely remove,^41^ we attempted to devise and apply mimetic OINCs with the aim of inducing CD301b^+^ macrophages by absorbing inflammatory factors and controlled IL-4 release. The AuNC component had a drug-carrying and controlled release effect, but a previous study has also shown that AuNC may act as an inducer of M2 macrophages, and could reduce tissue inflammation.^42^ Proinflammatory cytokines such as IL-1β and TNF-α play prominent roles in disturbing the Wnt and BMP signaling pathways, leading to aberrant osteoblast activity.^43,44^ More importantly, blocking the proinflammatory cytokines IL-1β and TNF-α is a prerequisite for successful induction of CD301b^+^ macrophages.

OINCs inherit the antigenic profile of neutrophils (IL-1R, TNF-αR), enabling them to act as decoys that can absorb and block IL-1β and TNF-α, coupled with IL-4 release controlled by 690-nm far-red irradiation, collectively promoting the induction and enrichment of CD301b^+^ macrophages in periodontal tissue, thereby promoting periodontal osteogenic activity. OINCs achieved desirable results in vivo, but IL-4 in OINCs could also induce M2 macrophages to an extent. The lack of a better strategy to specifically induce CD301b^+^ macrophages made it possible that the function of CD301b^+^ macrophages in in vivo application may be partially influenced by M2 macrophages.

In conclusion, we identified a novel CD301b^+^ macrophage population accumulated in periodontal tissue that effectively boosts periodontal bone regeneration. Unlike traditional inflammation-regulating subsets, CD301b^+^ macrophages could be accurately defined as regeneration-related macrophages. Mechanistically, CD301b^+^ macrophages promoted osteogenic differentiation of BMSCs via IGF-1/Akt/mTOR signaling. Lastly, OINCs were designed to reduce periodontal inflammation and induce CD301b^+^ macrophage differentiation, thereby improving periodontal osteogenic activity. The current study revealed a new mechanism of periodontal bone regeneration from the perspective of CD301b^+^ macrophages, and an intervening strategy for periodontitis was developed. It is hoped that the method can be adapted as a promising therapeutic strategy for other inflammatory bone diseases.

## Materials and methods

### Ethical approval and mouse model

8-week-old female C57BL/6 mice were obtained from the Vital River company (Beijing, China). *Mgl2*^DTR^ mice were obtained from the Jackson Laboratory. Animals were bred and maintained under specific pathogen-free conditions. All procedures were conformed to the ARRIVE guidelines 2.0 and approved by the Animal Care and Use Committee of Shandong First Medical University & Medical Science Technology Innovation Center and Wuhan University, China (MLIC2021175).

As previously described, a bacterially retentive 5-0 silk ligature was installed between the first and second molars for inducing periodontitis.^45^ The contralateral side in each mouse was left untreated to serve as the control for bone loss calculation and baseline for other related assays. To explore the bone formation during the resolving phase of periodontitis, ligatures were removed on day 8 and the mice were monitored and analyzed for another 3, 6, and 10 days (denoted as 3DR, 6DR, 10DR). While in the other queue, ligatures remained in place for 8, 11, 14, and 18 days (denoted as 8DL, 11DL, 14DL, 18DL). At different timepoints, animals were sacrificed for further examinations. To observe the bone healing after CD301b^+^ macrophages depletion during the resolving phase of periodontitis, 100 μL PBS with or without 500 ng DT was injected intraperitoneally (i.p.) every one to two days per *Mgl2*^DTR^ mice at certain timepoint.

### Micro-computed tomography

Maxillary bones were harvested and fixed in 4% formaldehyde for 24 hours. Then specimens were scanned with the μCT system (SkyScan, Buker). In the reconstructed maxillary, the distance between the cementoenamel junction and alveolar bone crest (CEJ-ABC) was measured on the buccal of the ligated site (the distal buccal root of the first molar and mesial buccal root of the second molar) as previously described.^45^ To calculate bone loss, the CEJ-ABC distance of each ligated site was subtracted from the CEJ-ABC distance of the contralateral untreated site.^46^ The alveolar bone between the first and second molars was measured and the parameters of bone loss, BV/TV, Tb.Th., and Tb.Sp. were calculated.

### Tissue preparation and histological staining

The fixed maxillae were decalcified for 3 weeks at 4 °C. For frozen sections, the samples were dehydrated in 15%, 30%, and 60% sucrose solution for 12 h at 4 °C respectively and embedded in OCT (Sakura, America), followed by cryosection (8 μm). As for paraffin sections, the periodontal blocks were dehydrated in ethanol and xylene with gradients of different concentrations and then embedded in 5 µm thick paraffin sections. The sections were stained with (1) H&E, (2) and TRAP with the Leukocyte Acid Phosphatase Kit (Sigma-Aldrich). The staining procedures were performed according to the manufacturer’s instructions. Immunostaining was used to detect the expression of CD301b^+^ macrophages and osteogenic-associated indicators. The primary antibodies were used: rat anti-CD301b (1: 100, Invitrogen, Cat No: 14-3011-95), goat anti-CD206 (1: 200, R&D Systems, Cat No: AF2535), goat anti-ALP (1: 200, R&D Systems, Cat No: AF2910), rabbit anti-OSX (1: 200, Abcam, Cat No: ab22552), mouse anti-IL-1β (1: 200, CST, Cat No: 12242), rabbit anti-TNF-α (1: 200, CST, Cat No: 3707). For immunohistochemistry, the sections were incubated with the secondary antibody and immersed in a DAB solution for coloration, followed by counterstaining with hematoxylin. For immunofluorescence, slides were incubated with secondary fluorescent antibodies (1:200, Invitrogen) and DAPI. Slides were observed and captured using the orthographic microscope (Olympus, Japan). The percentage of positive area was analyzed by ImageJ software.

### CD301b^+^ macrophage induction in vitro

BMDMs were extracted from the femur and tibia bone marrow of C57BL/6 mice. Bone marrow cells were plated and cultured in the presence of recombinant murine macrophage colony-stimulating factor (M-CSF; 20 ng·mL^−1^, PeproTech). Five days later, the mature BMDMs were eligible for subsequent experiments. BMDMs were stimulated with IL-4 (20 ng·mL^−1^, PeproTech) for 24 h. The induction efficiency of CD301b^+^ macrophages was determined by flow Cytometry. To detect the effect of an inflammatory environment on the induction of CD301b^+^ macrophages, CD301b^+^ macrophages were detected by immunofluorescence staining after co-incubation with IL-4 in the presence of IL-1β (20 ng·mL^−1^, PeproTech) and TNF-α (20 ng·mL^−1^, PeproTech).

### Flow cytometry and sorting

Given the small amount of periodontal soft tissue, gingival tissue from 3 mice taken at the same timepoint was pooled and analyzed as one sample. Excised periodontal tissues were minced and digested with RPMI-1640 containing collagenase II (2 mg·mL^−1^, Thermo Fisher Scientific Inc.) and collagenase IV (2 mg·mL^−1^, Thermo Fisher Scientific Inc.) for 1 h, then single-cell suspensions were obtained. The antibodies used were CD16/CD32 (1:200, BD Biosciences, Clone: 2.4G2), CD45-APC/Cyanine7 (1:400, Biolegend, Cat No. 103116), CD11b-Pacific Blue (1:200, Biolegend, Cat No. 101223), F4/80-APC (1:400, Biolegend, Cat No. 123115), CD301b-PE (1:200, Biolegend, Cat No. 146803), CD206-FITC (1:200, Biolegend, Cat No. 141703), and Ly6C-PE/Cyanine7 (1:400, Biolegend, Cat No. 128017). Cell viability was determined with Fixable Viability Stain 510 (1:500, BD Biosciences, Cat No. 564406). Flow cytometry analysis of stained cells was conducted using an LSR FortessaX20 (BD, USA).

For periodontal macrophage sorting, periodontal tissue acquired from 6DR models was processed in accordance with the above-described procedure, and CD301b^-^ macrophages and CD301b^+^ macrophages were sorted using a FACS Aria II (BD Bioscience). Cells (*n* = 2 replicates per group) were collected in 1.5-mL centrifuge tubes for SMART RNA sequencing. For in vitro CD301b^+^ macrophage sorting, after 24 hours of IL-4 induction (20 ng·mL^−1^, PeproTech), BMDMs were harvested and prepared as single-cell suspensions. Similar to periodontal macrophage sorting, BMDM-derived CD301b^−^ macrophages and CD301b^+^ macrophages were obtained and cultured in α-MEM complete medium, and the supernatant was collected every two days for a total of four days as CM for inducing mineralization. CM derived from untreated BMDMs served as the M0 control group.

### Osteogenesis assays

BMSCs were obtained from the femur and tibia bone marrow of C57BL/6 mice. BMSCs at passage three were utilized for osteogenic mineralization assay. To verify the role of CD301b^+^ macrophages on osteogenesis, BMSCs were co-cultured with CM derived from different macrophage subtypes at the dilution ratio of 1:1 for osteogenic differentiation assay. The total RNA of BMSCs was extracted, and the osteogenic gene (*alp, runx2, col1*) expression was detected on day 7. ALP staining (Beyotime Biotechnology) and alizarin red staining (Sigma Aldrich) were performed according to the instructions on day 7 and day 14, respectively. Stained cells were observed and imaged using an optical microscope.

### RNA sequencing and quantitative real-time PCR

At 6DR, CD301b^-^ and CD301b^+^ macrophages were sorted from periodontal tissues and collected for SMART RNA sequencing (Illumina, USA). Expression levels were calculated using RSEM (v.1.3.1). Differential expression analysis was performed using DESeq2 (v.1.4.5). For all upregulated and downregulated DEGs, GO and GSEA analyses were performed using the free online Dr. TOM II Platform. The top seventy-eight upregulated DEGs associated with the process of osteogenic induction regulation were used for heat map analysis.

To monitor the level of periodontal inflammation, excised gingival tissue was used to extract total RNA in accordance with the instructions of the RNAprep Pure Micro Kit (TIANGEN). For cell experiments in vitro, total RNA was extracted via Trizol reagent in accordance with the manufacturer’s instructions (TAKARA, Japan). The RNA was reverse-transcribed using PrimeScript RT Master Mix (Takara, Japan) in accordance with the manufacturer’s instructions. Real-time PCR with cDNA was performed with a LightCycler 480 Instrument (Roche) using SYBR Green (Takara). Relative target mRNA expression normalized with *Gapdh* was determined. Data were analyzed using the comparative (2^-ΔΔct^) method. The primers used are shown in Table 1.

**Table 1:** Primer sequences used in RT-PCR

	Primer sequence	
Gene	Forward	Reverse
*Gapdh*	GACTGATGTTGTTGACAGCCACTG	TAGCCACTCCTTCTGTGACTCTAAC
*Il1β*	TGGACCTTCCAGGATGAGGACA	GTTCATCTCGGAGCCTGTAGTG
*Tnfα*	GGTGCCTATGTCTCAGCCTCTT	GCCATAGAACTGATGAGAGGGAG
*Alp*	CCTTGAGTCCTTGCGCGGCA	TTGGCCCTCCTCCTCCAGCC
*Runx2*	CACCACGCTCTTCTGTCTACTGAA	TGCCACAAGCAGGAATGAG
*Col1*	CCTCCTCAGCTCACCTTCTC	GTTGGGAGCCCAAATAGAAA
*Igf1*	GTGGATGCTCTTCAGTTCGTGTG	TCCAGTCTCCTCAGATCACAGC

### Western blot

For the extraction of total protein, 80 μl radio immunoprecipitation assay (RIPA) lysis buffer (Beyotime Biotechnology) containing protease inhibitor and 1% phosphatase inhibitor was used to lyse the cells. All samples were quantified and normalized by a bicinchoninic acid (BCA) protein assay kit (Thermo Fisher Scientific). After heated for 10 min at 95°C, the samples were separated via sodium dodecyl sulfate polyacrylamide gel electrophoresis (SDS-PAGE) and transferred to a polyvinylidene fluoride membrane (Millipore). The membranes were incubated with antibodies against IGF1R (1:1 000, ABclonal), p-IGF1R (1:1 000, ABclonal), Akt (1:1 000, ABclonal), p-Akt (1:1 000, Abcam), mTOR (1:1 000, CST), p-mTOR (1:1 000, CST), GAPDH (1:1 000, ABclonal) at 4 °C overnight, followed by horseradish peroxidase (HRP)-conjugated secondary antibodies (Sigma) treated for 1 h. Blots were visualized by the enhanced chemiluminescence (ECL) HRP Substrate Kit (Thermo Fisher Scientific, US).

### Preparation and characterization of OINC

Peripheral blood was collected from lipopolysaccharide-stimulated mice and neutrophils were collected by gradient centrifugation into 15-mL centrifuge tubes as previously described.^47^ At 4 °C, 35% amp ultrasound was applied for 5 s, then after a 3-s pause the procedure was repeated for 45 min. The suspension was centrifuged at 4 000 r·min^−1^ for 5 min, then the supernatant was collected and centrifuged at 20 000 *g* for 30 min in an ultracentrifuge at 4 °C. The supernatant was then collected and centrifuged at 70 000×*g* for 2 h. The precipitate was resuspended in ultra-pure water and the concentration of membrane protein was detected via a BCA protein assay kit (Thermo Fisher Scientific). AuNC was synthesized and IL-4 was loaded via a phase transition method as previously described,^23^ and together with extracted mouse neutrophil membranes they were assembled into OINCs by a micro-extruder (Avanti, USA). Transmission electron microscopy (JEM2100, Japan) was used to examine the morphologies of AuNCs and OINCs. Hydrodynamic diameters and zeta potentials were detected by dynamic light scattering. Ultra-violet-visible absorption spectra were observed and recorded by a dual-beam spectrophotometer (TU-1901, China) in the wavelength range of 400–900 nm to detect the absorption peaks of AuNCs and OINCs.

To investigate the in vitro cytotoxicity of AuNCs, RAW264.7 cells were incubated with AuNC solutions of different concentrations (0, 12.5, 25.0, 50.0, and 100.0 μg·mL^−1^) for 12 h, 24 h, and 48 h. Cell vitality was evaluated with a cell counting kit (CCK-8). To evaluate the capacity of OINCs to control drug release, OINCs were loaded with fluorescein methylene blue (MB) as a model drug, then 1 mL of OINCs was irradiated with a 690-nm wavelength far-red laser for 5 min, repeated 3 times. The irradiation power density was 3.0 W·cm^−2^. A fluorescence spectrometer was used to monitor the release kinetics of the fluorescein MB. In vitro, the photothermal effects of AuNCs and OINCs were detected using a far-red laser at 3.0 W·cm^−2^ for 15 min. In vivo, twenty-five microliters of solution containing AuNCs or OINCs was injected into the periodontal tissue between the first and second molars from the buccal and palatal sides, respectively. The normal saline group served as a control, and a 690-nm diode laser system (BWT Beijing Co., Ltd., Beijing, China) was used as an irradiation source (3.0 W·cm^−2^ power density). The FLIR A65sc test kit (FLIR Systems, Inc. USA) was used for temperature detection and thermal image recording.

### Functional verification of AuNCs and OINCs

Western blotting was used to investigate the protein expression of cytokine receptors on OINCs. Mouse neutrophils were collected in RIPA buffer containing protease inhibitor, quantified via a BCA protein assay kit (Thermo Fisher Scientific), and mixed with loading buffer. Neutrophil lysate and OINCs with comparable protein content were loaded into a 10% SDS-PAGE gel, and AuNCs served as a control. IL-1R (1:1 000, Santa Cruz) and TNF-αR (1:1 000, CST) were visualized by the ECL method. To evaluate the neutralization efficiency of OINCs with respect to IL-1β and TNF-α (PeproTech, USA), 100 μL OINCs (membrane protein concentrations 200, 500, and 1 000 μg·mL^−1^) were incubated with IL-1β (4 ng·mL^−1^) or TNF-α (4 ng·mL^−1^). The pellet was removed by centrifugation at 16 000 *g* for 30 min at 4 °C. Cytokines remaining in the supernatant were detected by ELISA (Bioswamp), and a 100 μL PBS-treated group was served as a control.

To evaluate the osteogenesis-promoting effects of OINCs on periodontitis, periodontitis was induced between the first and second molar of the maxilla in mice. Twenty-five microliters of solution containing AuNCs or OINCs was injected into the periodontal tissue between the first and second molars from the buccal and palatal sides, respectively, and a normal saline group served as a control. All injections in mice were performed under a stereomicroscope. A 690-nm wavelength far-red light was used on the day of injection and for 3 consecutive days thereafter to promote continuous release. To ensure sufficient drug release 4 times, we determined that an adequate time for each in vivo irradiation was 5 min. An additional injection was performed on the 4th day, the irradiation was the same as that utilized previously, and the mice were sacrificed on day 8. Samples were collected for μCT detection and histological examination.

### Statistics

Data were represented as means ± SEM of at least three independent experiments. Statistical analysis was performed with GraphPad Prism software (version 8.0). Significant differences were calculated with Student’s *t* test or by analysis of variance (ANOVA), followed by the Bonferroni post hoc test, for multiple comparisons. *P* < 0.05 was considered as a statistically significant difference. The symbols *, **, and *** indicate *p* values < 0.05, 0.01, and 0.001 respectively; ns. not significant.

## Supplementary information

IJOS202209524RR revised Supplementary information

## Data Availability

The data used and/or analyzed during the current study are contained within the manuscript. RNA sequencing data are available at the Sequence Read Archive (https://www.ncbi.nlm.nih.gov/sra) with BioProject accession: PRJNA914415. Other data are available from the corresponding author on reasonable request.
