# Supplementary material for: CD301b+ macrophage: the new booster for activating bone regeneration in periodontitis treatment
Source: Int J Oral Sci. 2023 May 17;15:19. doi: 10.1038/s41368-023-00225-4 (PMC10192316; doi:10.1038/s41368-023-00225-4)
Supplement: Supplementary file 1 — IJOS202209524RR revised Supplementary information [file 41368_2023_225_MOESM1_ESM.docx]

**CD301b^+^ macrophage: the new booster for activating bone regeneration in periodontitis treatment**

Can Wang^1^†, Qin Zhao^1^†, Chen Chen^1^, Jiaojiao Li^1^, Jing Zhang^1^, Shuyuan Qu^1^, Hua Tang^2^, Hao Zeng^1^, Yufeng Zhang^1, 3 *^

**
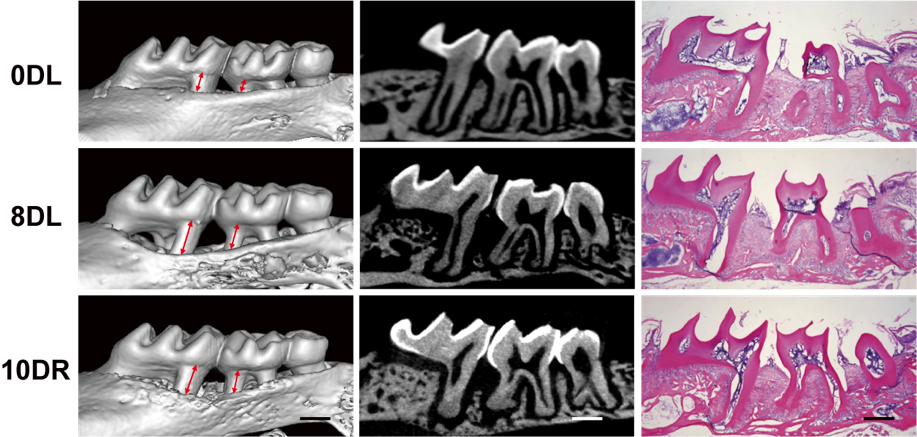
**

**Figure S1. Representative images to observe periodontal bone level change**

μCT analysis and H&E staining showed that bone level (bidirectional red arrows) changes in the 0DL, 8DL, and 10DR groups. Scale bar, 500 μm.

**
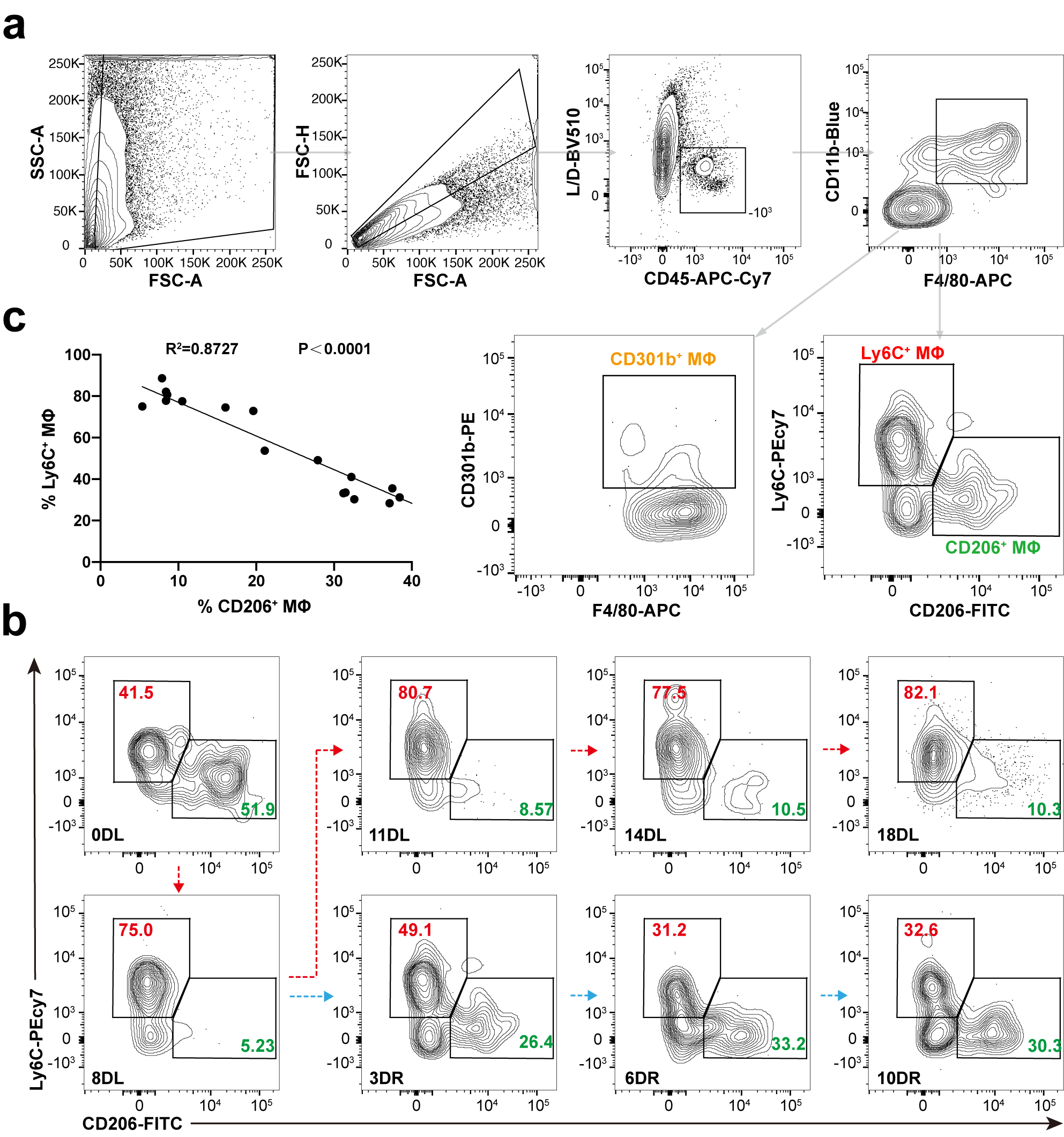
**

**Figure S2.** **The variation profile of pro-inflammatory and anti-inflammatory macrophage in inflammatory and healing phase of periodontitis**

**a** Gating strategy of macrophages from periodontal tissue through flow cytometry analysis. **b** Representative flow cytometry contour plots of Ly6C^+^ or CD206^+^ macrophages at different time points in the ligature persistence or removal phase. **c** Correlation between the percentage of Ly6C^+^ and that of CD206^+^ macrophages at different timepoints.

**
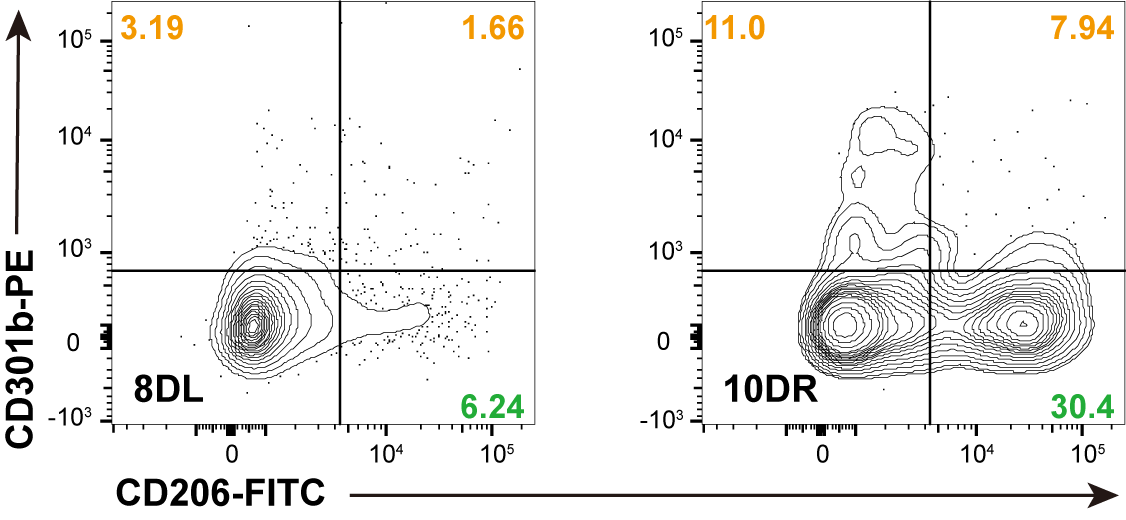
**

**Figure S3. Detection of CD301b^+^ macrophages with CD206^+^ macrophages by flow cytometry**

Representative flow cytometry diagram of macrophages clustered by CD301b and CD206

**
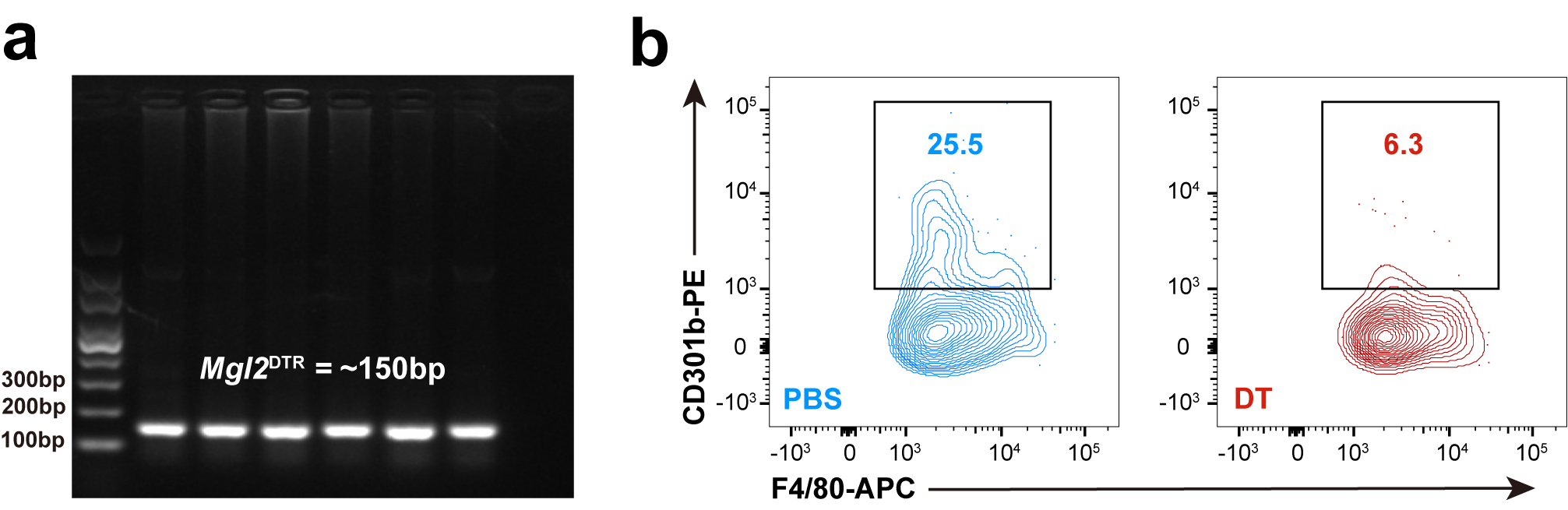
**

**Figure S4. Genotype identification of *Mgl2*^DTR^ mice and efficiency of CD301b^+^ macrophage depletion**

**a** Genotyping assay showed that the band of mutant mice (*Mgl2*^DTR^) was located at ~150bp. **b** Validation of CD301b^+^ macrophage depletion efficiency by flow cytometry at 6DR.

**
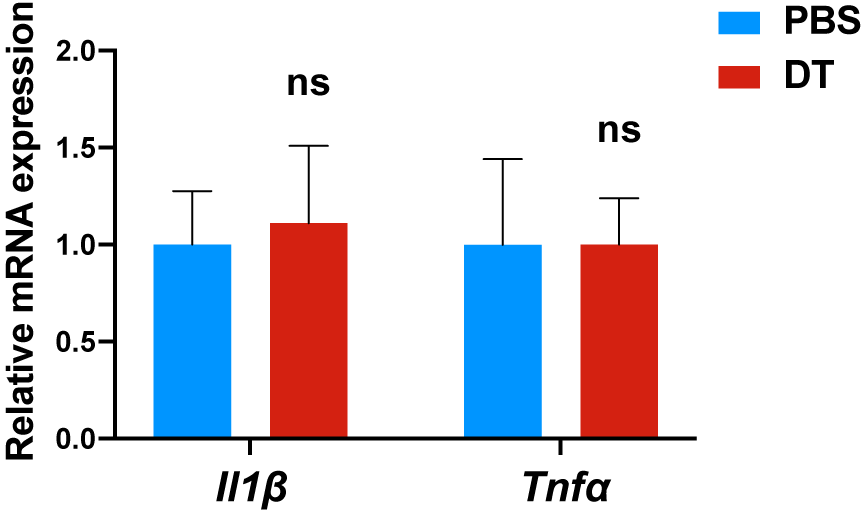
**

**Figure S5. RT-PCR detection of *Il1β and Tnfα* expression level of the DT and PBS group.**

Ten days after ligature removal (10DR) the expression levels of *Il1β* and *Tnfα* in the periodontal tissue of the two groups were detected by RT-PCR analysis. Data were normalized to *Gadph* and are presented as fold change relative to the PBS group (*n* = 4).

**
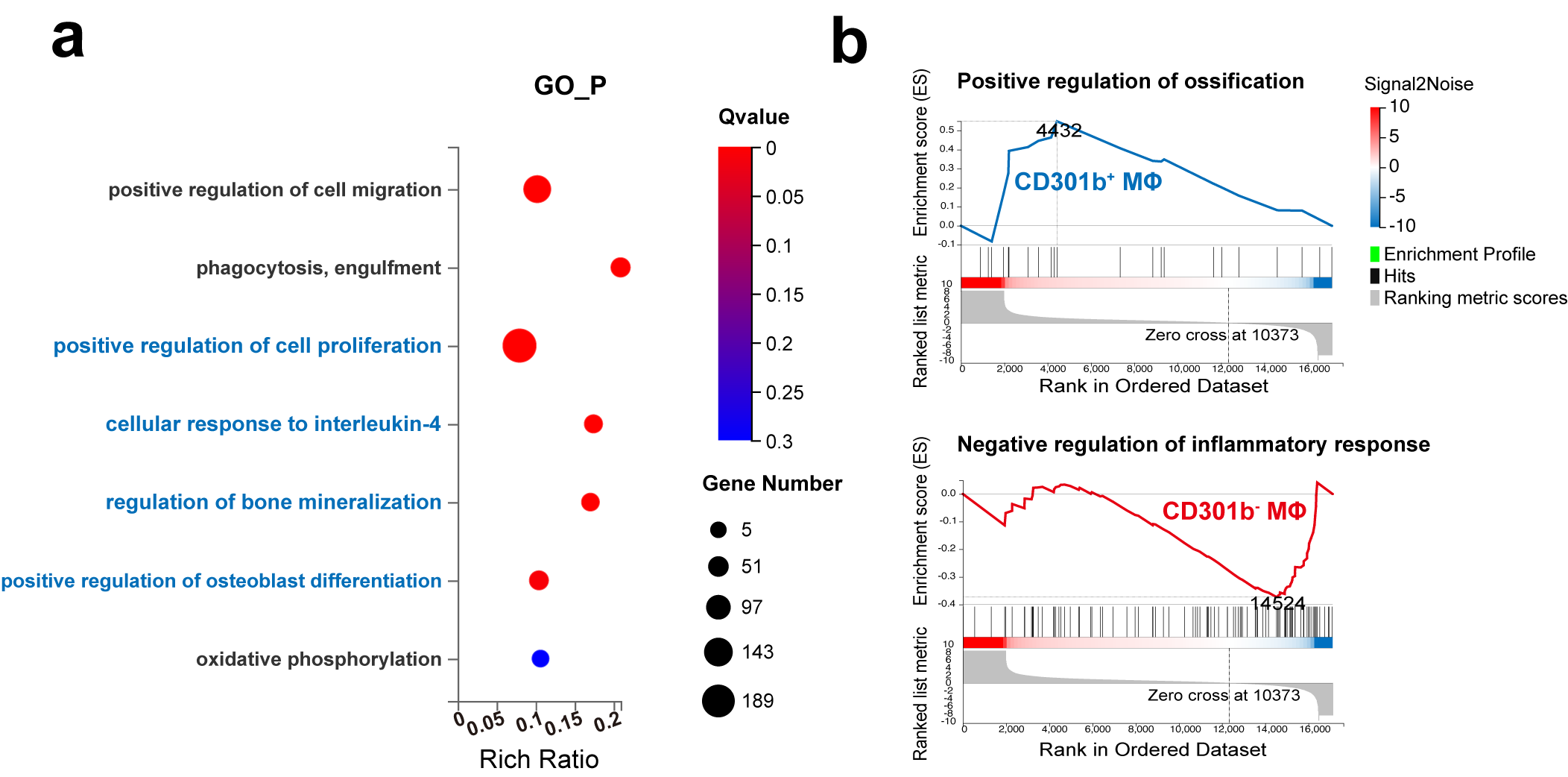
**

**Figure S6. GO enrichment analysis and GSEA of differentially expressed genes from CD301b^+^ macrophages and CD301b^-^ macrophages**

**a** GO analysis of DEGs in CD301b^+^ macrophages compared with CD301b^-^ macrophages. **b** GSEA analysis comparing CD301b^+^ macrophages and CD301b^-^ macrophages. Plots with higher peaks (above) indicate enrichment of gene sets in CD301b^+^ macrophages, plots with negative peaks (below) indicate enrichment of gene sets in CD301b^-^ macrophages.

**
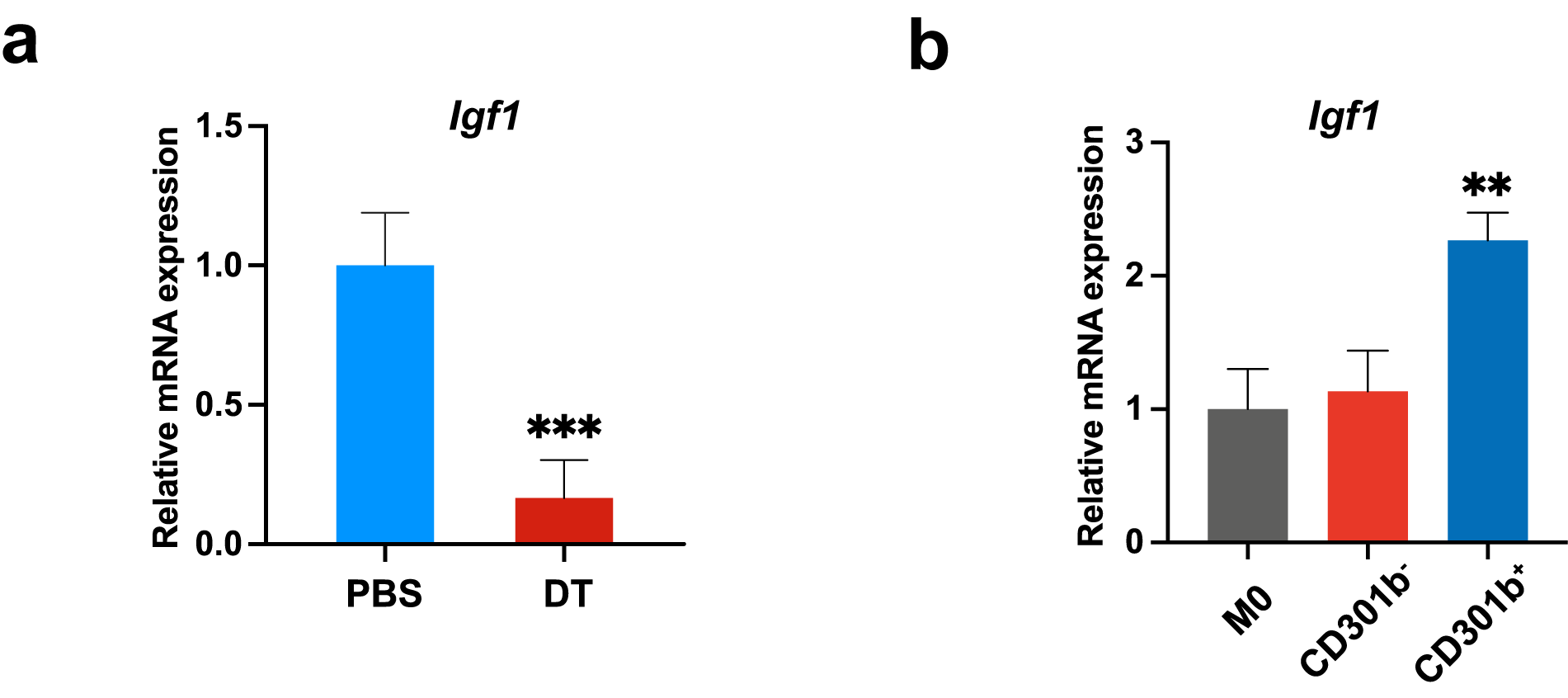
**

**Figure S7. RT-PCR detection of *igf1* expression level *in vivo* and *in vitro***

**a** *Igf1* expression level in periodontal tissue (10DR) of CD301b^+^ macrophages ablation mice (n = 3). **b** RT-PCR to detect the *igf1* expression level of M0, CD301b^-^ and CD301b^+^ macrophages induced and sorted *in vitro* (*n* = 3).


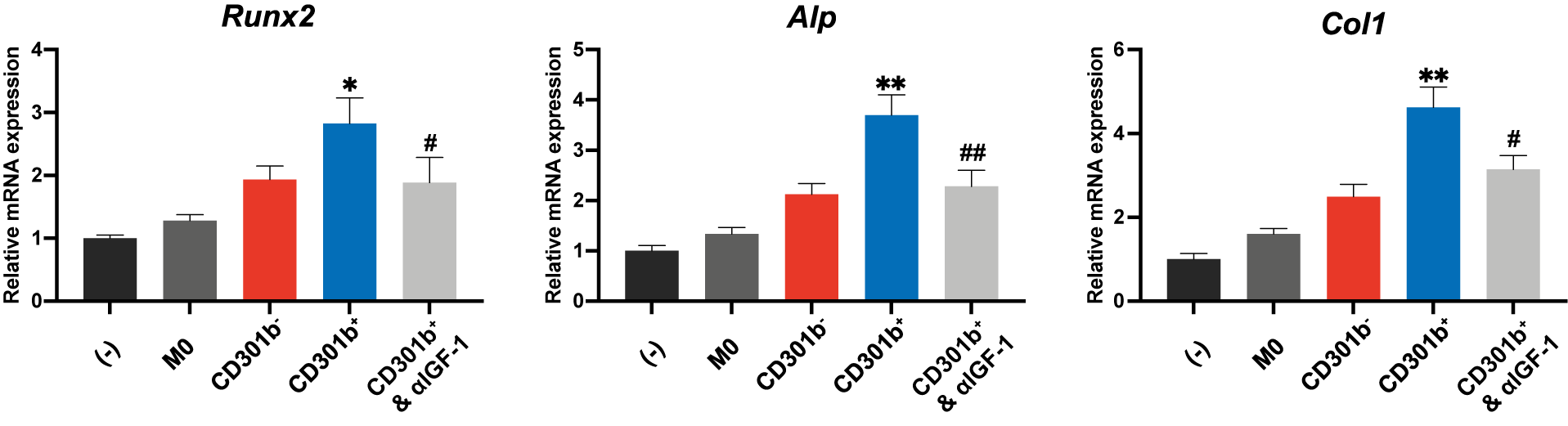


**Figure S8. RT-PCR detection of osteogenesis-related genes expression level.**

Relative mRNA expression of *runx2*, *alp*, and *col1* in BMSCs with different treatments (*n* = 3).


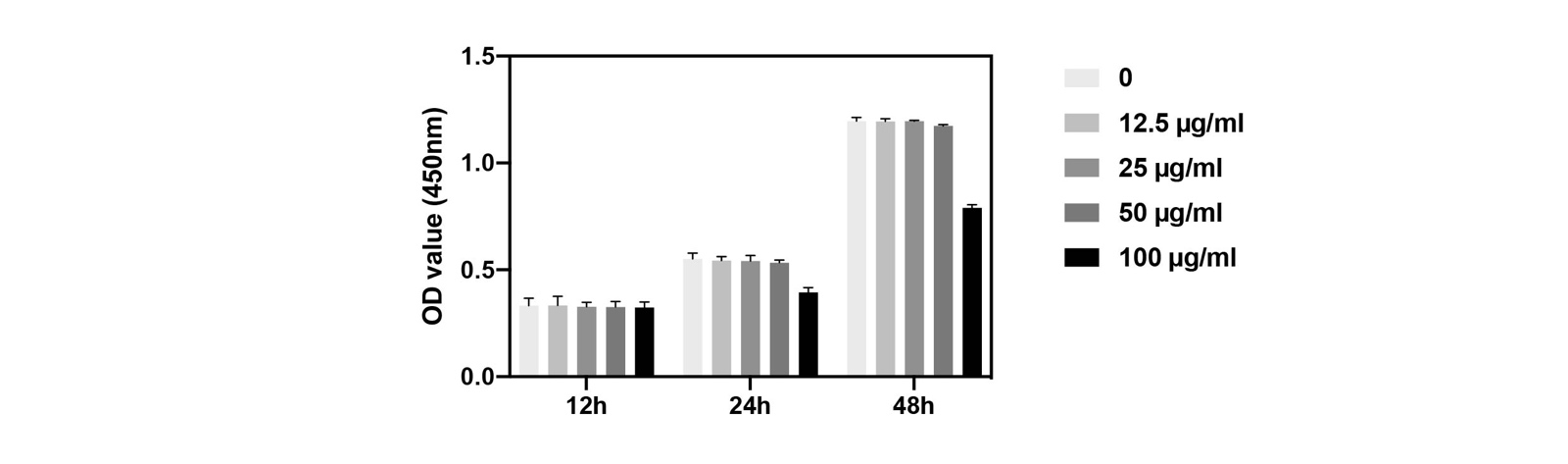


**Figure S9. Cell viability at different concentrations of AuNCs**

RAW264.7 cell line viability evaluated by CCK-8 assay after incubation with different concentrations of AuNC for 12 h, 24 h and 48 h (*n* = 3).


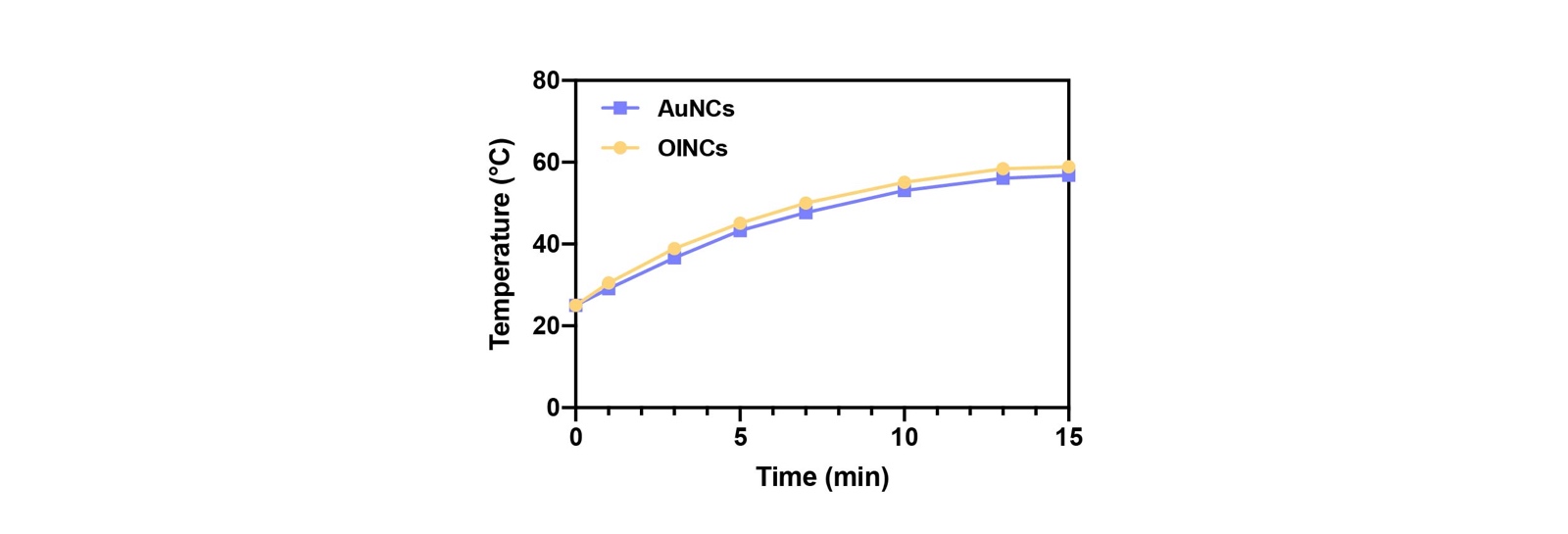


**Figure S10. Photothermal curve of AuNCs and OINCs**

Temperature-time curve of AuNCs and OINCs under 690-nm far-red irradiation at 3.0 W cm^-2^ power density.
